# Anti-pentraxin 3 antibodies and residual disease activity in rheumatoid arthritis

**DOI:** 10.1093/rheumatology/keae529

**Published:** 2024-09-28

**Authors:** Mariangela Salvato, Francesca Frizzera, Anna Ghirardello, Antonia Calligaro, Costantino Botsios, Margherita Zen, Andrea Doria, Alessandro Giollo

**Affiliations:** Rheumatology Unit, Department of Medicine, University of Padova, Padova, Italy; Rheumatology Unit, Department of Medicine, University of Padova, Padova, Italy; Rheumatology Unit, Department of Medicine, University of Padova, Padova, Italy; Rheumatology Unit, Department of Medicine, University of Padova, Padova, Italy; Rheumatology Unit, Department of Medicine, University of Padova, Padova, Italy; Rheumatology Unit, Department of Medicine, University of Padova, Padova, Italy; Rheumatology Unit, Department of Medicine, University of Padova, Padova, Italy; Rheumatology Unit, Department of Medicine, University of Padova, Padova, Italy

**Keywords:** RA, PTX3, pentraxin, DMARDs, biological, pain, patient global assessment, CDAI

## Abstract

**Objectives:**

This study quantified anti-PTX3 antibodies in the serum of seropositive and seronegative RA patients, examining their associations with disease activity and patient-reported outcome measures (PROMs).

**Methods:**

In this cross-sectional study, RA patients diagnosed per ACR/EULAR 2010 criteria were recruited. Seronegative RA was defined as ACPA <7 kU/L. Data on demographics, clinical characteristics, medications, and PROMs were collected. Serum anti-PTX3 antibodies were measured using an in-house ELISA method. Comparative analyses were conducted with historical controls having PsA and FM.

**Results:**

The cohort included 83 RA patients (42 seropositive, 41 seronegative). Seropositive patients had lower anti-PTX3 antibody levels than PsA (*P* = 0.001) and FM (*P* = 0.004) controls. Seronegative patients had higher levels than seropositive ones (*P* = 0.032). Anti-PTX3 antibodies correlated with CDAI (*r* = 0.255), PtGA (*r* = 0.257), VAS-GH (*r* = −0.235), VAS-pain (*r* = 0.233), and HAQ (*r* = 0.311), but not with joint counts, inflammatory markers, or physician’s global assessment. The PtGA association remained significant when adjusted for BMI, SJC28, ESR, and prednisone dosage (β = 0.206, *P* = 0.042). Patients with near-controlled RA (SJC28 ≤ 2, PtGA > 2) had higher anti-PTX3 levels than those with controlled disease (SJC28 ≤ 2, PtGA ≤ 2; *P* = 0.048). Tocilizumab or abatacept-treated patients had lower levels compared with those on TNFi or JAKi.

**Conclusion:**

Elevated anti-PTX3 antibodies in RA indicate residual active disease despite controlled inflammation. They may serve as a biomarker for true active disease, especially in seronegative RA patients who might be undertreated.

Rheumatology key messages

- Elevated anti-PTX3 antibodies indicate residual active disease in RA despite controlled inflammation.
- Anti-PTX3 antibodies correlate with patient-reported outcome measures in RA, especially seronegative patients.
- Lower anti-PTX3 levels are observed in RA patients treated with tocilizumab or abatacept.

## Introduction

RA is an autoimmune disorder associated with several autoantibodies. While the most known are RF or ACPA, other autoantibodies have been discovered, though clinical associations have been weak, and thus there has never been a clinical purpose for testing such autoantibodies.

One of the most intriguing discoveries in autoimmunity are anti-pentraxin-3 (PTX3) antibodies, which have been detected in up to a third of RA patients who do not express ACPA (i.e. seronegative RA) [1]. Such antibodies target and suppress the action of PTX3, which is the prototype of long pentraxins, a superfamily of soluble pattern recognition receptors of innate immunity involved in the acute phase response to infection, inflammation, tissue remodelling, and female fertility [2]. PTX3 synthesis is mainly extrahepatic, reflecting a local inflammatory response. PTX3 is stored in neutrophil lactoferrin-containing granules, from which it is quickly released in response to microbial and inflammatory triggers and co-localizes in neutrophil’s extracellular traps (NETs) [3, 4]. Furthermore, several cell lineages, including myeloid, epithelial, endothelial, and fibroblasts, rapidly synthesize PTX3 in response to inflammatory stimuli such as cytokines, toll-like receptor (TLR) agonists, and microbial moieties. In contrast, PTX3 expression is inhibited by IFN-ɣ, glucocorticoids, vitamin D3, PGE2, and IL-4/IL-1. PTX3 can bind a great number of ligands, including complement components, growth factors, extracellular matrix glycoproteins, microbes, and apoptotic cells, suggesting a role as a modulator molecule at the intersection of inflammatory and anti-inflammatory response in both physiologic and pathologic conditions. In RA, there is clear documentation of the role of PTX3 in synovial [5, 6] and systemic [7] inflammation, association with ACPA/RF [6], disease activity and bone erosions [5, 8, 9]. However, whether the presence of anti-PTX3 antibodies correlates with disease activity in RA is unknown.

Clinical and preclinical data suggest that anti-PTX3 antibodies have anti-inflammatory and immunomodulatory effects in SLE [10–15], and ANCA-associated vasculitis (AAV) [16, 17]. Herein, we sought to clinically confirm that anti-PTX3 antibodies have similar effects in RA as well. The expected result was to detect higher levels of anti-PTX3 antibodies in RA patients with higher disease activity. However, we were also interested in understanding whether anti-PTX3 antibodies were expressed in ACPA-negative RA patients. For this reason, we enriched our study population with seronegative RA patients.

## Materials and methods

### Study population and design

We performed a single-centre, exploratory, cross-sectional study. Consecutive patients attending the University and Hospital of Padua’s outpatient clinic from February 2022 to September 2022 were asked to participate in the study. The inclusion criteria were age ≥18 years, the signature of informed consent, clinical diagnosis of RA, and classification according to the 2010 ACR/EULAR criteria for RA. Experienced rheumatologists (A.G., M.Z., A.C. and C.B.) confirmed the diagnosis of RA based on clinical examination, laboratory findings, disease history, and imaging. Patients with a more likely alternative diagnosis (PsA, spondyloarthritis, crystal-induced arthropathy, osteoarthritis, FM, or infectious arthritis) were excluded.

We also included the cohorts of patients with FM and PsA from our seminal paper in 2010 that used the same method to detect anti-PTX3 antibodies in SLE and across a spectrum of rheumatic and musculoskeletal conditions [10]. Clinical data and the medications of those participants were not available.

### Data collection

Sex, age, weight, height, smoking status, clinical (TJC28, SJC28), laboratory (ESR, CRP, complete blood count), and treatment variables were recorded. The use of glucocorticoids and the mean prednisone-equivalent dose, as well as current and previous treatment with csDMARDs (MTX), leflunomide, sulphasalazine, and hydroxychloroquine), and b/tsDMARDs (TNF inhibitors, IL-6 inhibitors, CTLA4 inhibitor, CD20 inhibitor, IL-1 inhibitor, and JAK inhibitors), were registered. Seropositivity for RF (<14 kU/L) and/or anti-citrullinated peptide activities (ACPA <7 kU/L), presence of radiographic erosions, and extra-articular features of RA were retrieved from patient records. Extra-articular manifestations included skin (rheumatoid nodules, cutaneous vasculitis, and neutrophilic dermatosis), pulmonary (interstitial lung disease, lung nodules, pleuritis), eye (uveitis, scleritis, Sjogren disease), haematological (Felty syndrome, large granular lymphocytic leukaemia), cardiac (pericarditis, myocarditis), neurological (peripheral neuropathy, carpal tunnel syndrome), and systemic (fever, weight loss) manifestations.

### Assessment of disease activity

Disease activity was measured by the DAS28-CRP, DAS28-ESR, Patient Global Assessment (PtGA), Physician Global Assessment (PhGA), Clinical Disease Activity Index (CDAI), and Simplified Disease Activity Index (SDAI) at baseline and after 6 months of follow-up. Remission was defined as a CDAI ≤ 2.8. Disease activity was categorized into three levels: low, moderate, and high according to CDAI cut-offs. The decision to use CDAI as the basis for remission and disease activity classification was motivated by its stringent nature. CDAI is widely acknowledged as a reliable measure for defining remission, and achieving remission according to CDAI is associated with better outcomes. The following validated PROMs (patient-reported outcome measures) were evaluated: PtGA, Patient Pain (VAS-pain), Patient Global Health (VAS-GH), HAQ-Disability Index (HAQ-DI), and Functional Assessment of Chronic Illness Therapy-Fatigue Scale (FACIT-Fatigue).

### Measurement of anti-PTX3 antibodies

Blood samples were collected from the antecubital vein and stored at −80°C. The measurement of anti-PTX3 antibodies was performed in serum using a standardized in-house ELISA method previously validated in a larger population of patients with connective tissue diseases, including a group with seropositive RA [10]. Recombinant PTX3 was obtained by purification from transfected Chinese Hamster Ovary (CHO) cells. Nunc MaxiSorp™ immunoplates (Nalge Nunc, New York, USA) were coated with 50 µl/well of human recombinant PTX3 diluted in PBS (pH 7.4). at a concentration of 5 µg/ml and incubated overnight at 4°C. The wells were blocked with 3% BSA (Sigma)/PBS and incubated at room temperature for 2 h. After three washes with the non-ionic detergent PBS/0.05% Polysorbate 20 (Tween-20, Sigma), sera were added in duplicate diluted 1:200 in 1% BSA/PBS and incubated at room temperature for 4 h. After three washes with washing buffer, alkaline phosphatase-conjugated anti-human immunoglobulin G (IgG) (γ-chain-specific) (Sigma, St Louis, Missouri, USA) 1:10000 in 1% BSA/PBS was added and incubated for 1 h at 37°C. Finally, after three washes, p-nitrophenyl phosphate (Sigma) tablets were added to a 10 ml substrate solution (magnesium carbonate, pH 9.6). The anti-PTX3 antibody titre was expressed as optical density (OD) at 405 nm. A positivity cut-off was previously determined by receiving operating characteristic curve analyses in which sensitivity was calculated in 130 patients with SLE and specificity in 130 healthy subjects at a value of 0.234 (sensitivity 46.2% and specificity 92.8%).

### Statistical analysis

We conducted univariate analyses to compare the clinical characteristics between seropositive (SPRA) and seronegative (SNRA) RA groups. Categorical variables were presented as absolute numbers and relative percentages, while continuous variables were reported as mean ± S.D. or median (interquartile range), depending on their distribution pattern. The Shapiro–Wilk test was utilized to evaluate normality. Associations between anti-PTX3 status and categorical variables were analysed using the chi-squared test or Fisher’s exact test, as appropriate. Continuous variables were analysed using the Student’s *t*-test or the Mann–Whitney *U* test. For comparisons involving more than two groups, analysis of variance (ANOVA) was employed with correction for multiple comparisons. Correlations between anti-PTX3 titres and variables of interest were examined through Pearson’s *r* test; non-linear predictors were natural-log transformed before running the analysis. Multivariable linear regression was conducted to examine the relationship between anti-PTX3 titres and PtGA. Covariates of interest were selected based on a literature review and those significantly associated with the outcome in univariable analyses. Statistical significance was defined as a *P*-value <0.05. All analyses were performed using IBM SPSS Statistics version 26 (USA) and GraphPad Prism 9.

## Results

### Characteristics of the study population

After applying inclusion and exclusion (*n* = 1) criteria, we enrolled consecutively 83 RA patients, of whom 42 were seropositive and 41 were seronegative. Table 1 compares the clinical features of seropositive and seronegative RA patients. The groups were well balanced for most characteristics, encompassing a common RA population of mostly female patients with a long disease duration. Half of the patients were in moderate or high disease activity according to CDAI cut-offs. Most patients in both groups were treated with b/tsDMARDs and MTX. Compared with seropositive RA, seronegative RA patients had significantly higher BMI, pain scores, tender joint counts, PtGA, and longer morning stiffness duration and received a greater dose of oral glucocorticoids.

**Table 1.** Demographics, disease activity and PROMs in 83 RA patients according to serostatus

Parameter	All RA	SPRA	SNRA	*P*-value
	(*N* = 83)	(*N* = 42)	(*N* = 41)	
Female sex	71 (85.4)	37 (88.1)	35 (85.4)	0.714
Age, years	62.8 ± 10.4	63.7 ± 10.3	61.9 ± 10.6	0.426
BMI	24.7 ± 4.5	23.6 ± 4.2	25.7 ± 4.7	**0.034**
Smoking status				
Current	12 (16.7)	8 (20.5)	4 (12.1)	0.481
Past	15 (20.8)	9 (23.1)	6 (18.2)	
Never	45 (62.5)	22 (56.4)	23 (69.7)	
Disease duration, years	16.0 ± 11.1	17.1 ± 11.8	14.8 ± 10.5	0.661
Erosions	56 (67.5)	30 (83.3)	26 (72.2)	0.257
Extra-articular manifestations	25 (30.1)	8 (19.5)	18 (43.9)	**0.018**
RF positive	38 (45.8)	41 (100)	1 (2.4)	**<0.001**
RF, kU/L	133.8 ± 239.9	256.8 ± 44.6	7.8 ± 1.3	**<0.001**
ACPA positive	39 (47.0)	42 (100)	0 (0)	**<0.001**
ACPA, kU/L	316.4 ± 598.9	621.8 ± 111.7	3.6 ± 0.5	**<0.001**
CRP, mg/L	6.8 ± 19.1	9.2 ± 23.5	4.5 ± 13.5	0.226
ESR, mm/h	20 ± 21	17.1 ± 17.5	22.8 ± 23.6	0.204
Anti-PTX3, OD	0.135 ± 0.111	0.117 ± 0.055	0.156 ± 0.101	**0.032**
**Disease activity**				
TJC, 0–28	3.4 ± 4.8	3.3 ± 5.1	3.4 ± 4.5	**0.012**
SJC, 0–28	1.6 ± 2.8	2.0 ± 3.3	1.1 ± 2.1	0.111
PhGA, 0–100	32.3 ± 28.6	26.9 ± 27.6	37.8 ± 28.8	0.082
DAS28 CRP	2.4 ± 1.1	2.3 ± 1.2	2.5 ± 1.1	0.300
DAS28 ESR	2.8 ± 13.3	2.9 ± 1.4	2.7 ± 1.2	0.566
CDAI	12.1 ± 11.3	11.6 ± 12.0	12.6 ± 10.6	0.187
Remission (≤2.8)	22 (26.5)	15 (35.7)	7 (17.1)	0.266
Low (>2.8 ≤ 10)	15 (18)	10 (23.8)	14 (34.1)	
Moderate (>10 ≤ 22)	32 (38.6)	10 (23.8)	13 (31.7)	
High (≥22)	14 (16.9)	7 (16.7)	7 (17.1)	
SDAI	12.8 ± 12.2	12.1 ± 12.7	13.6 ± 11.9	0.580
**PROMs**				
PtGA, 0–100	38.8 ± 32.0	36.1 ± 34.0	41.8 ± 30.0	**0.031**
PtGH, 0–100	57.5 ± 22.5	61.8 ± 18.7	52.1 ± 25.7	0.076
VAS-pain, 0–100	39.1 ± 30.9	35.2 ± 32.4	43.3 ± 29.1	0.237
FACIT-Fatigue, 0–52	35.8 ± 10.2	35.3 ± 10.5	36.2 ± 10.1	0.752
VAS-MS, 0–100	36.8 ± 33.0	37.7 ± 32.7	35.7 ± 33.8	0.801
HAQ-DI, 0–3	0.7 ± 0.7	0.7 ± 0.8	0.7 ± 0.6	0.975
**Current therapy**				
Glucocorticoids	33 (39.8)	13 (39.4)	20 (48.8)	0.097
Prednisone, mg daily	1.8 ± 2.7	1.8 ± 3.2	1.7 ± 2.2	**0.004**
csDMARDs	45 (54.2)	25 (55.6)	20 (48.8)	0.326
MTX	35 (42.2)	21 (50.0)	14 (40.0)	0.144
b/tsDMARD	63 (75.9)	31 (73.8)	32 (78.0)	0.652

All continuous data are reported as means ± S.D. All categorical variables are reported as absolute numbers (percentage). Values in bold are statistically significant.

TJC: tender joint count; SJC: swollen joint count; CDAI: Clinical Disease Activity Index; SDAI: Simplified Disease Activity Index; PtGA: Patient Global Assessment; PhGA: Physician Global Assessment; PtGH: patient global health; VAS: visual assessment scale; MS: morning stiffness; csDMARDs: conventional synthetic disease-modifying anti-rheumatic drugs; b/tsDMARDs: biologic or targeted synthetic disease-modifying anti-rheumatic drugs.

### Anti-PTX3 antibodies at RA population level

Anti-PTX3 antibodies were detected in all participants with a mean ± S.D. titre of 0.111 ± 0.107 OD nm.

**Figure 1.**
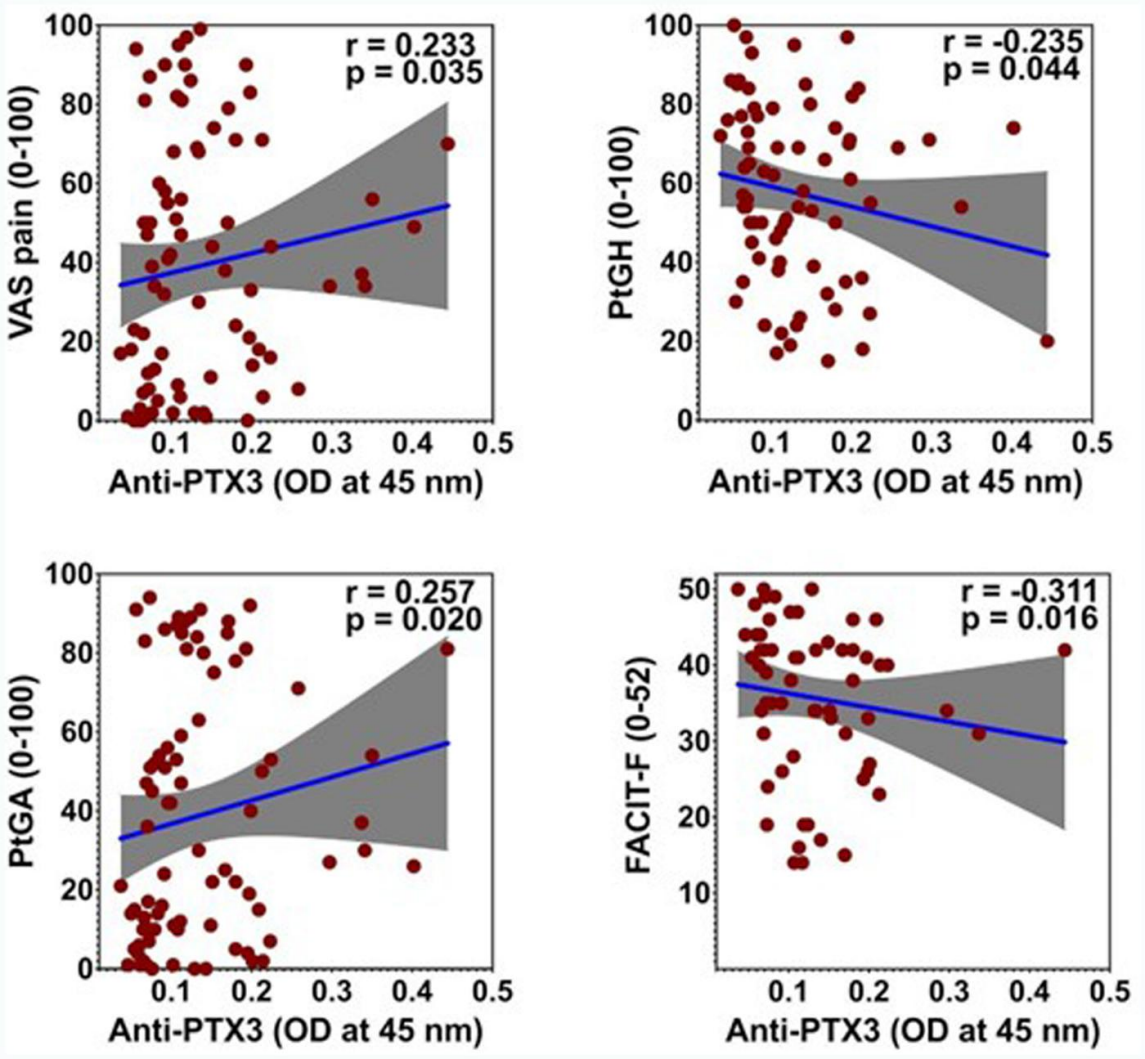
Correlations between anti-PTX3 antibody titre and disease outcomes. Cases are reported as dots, regression line, and 95% confidence interval for *r*. FACIT-Fatigue: Functional Assessment of Chronic Illness Therapy-Fatigue; PtGA: Patient Global Assessment; PhGA: Physician Global Assessment; PtGH: patient global health

**Figure 2.**
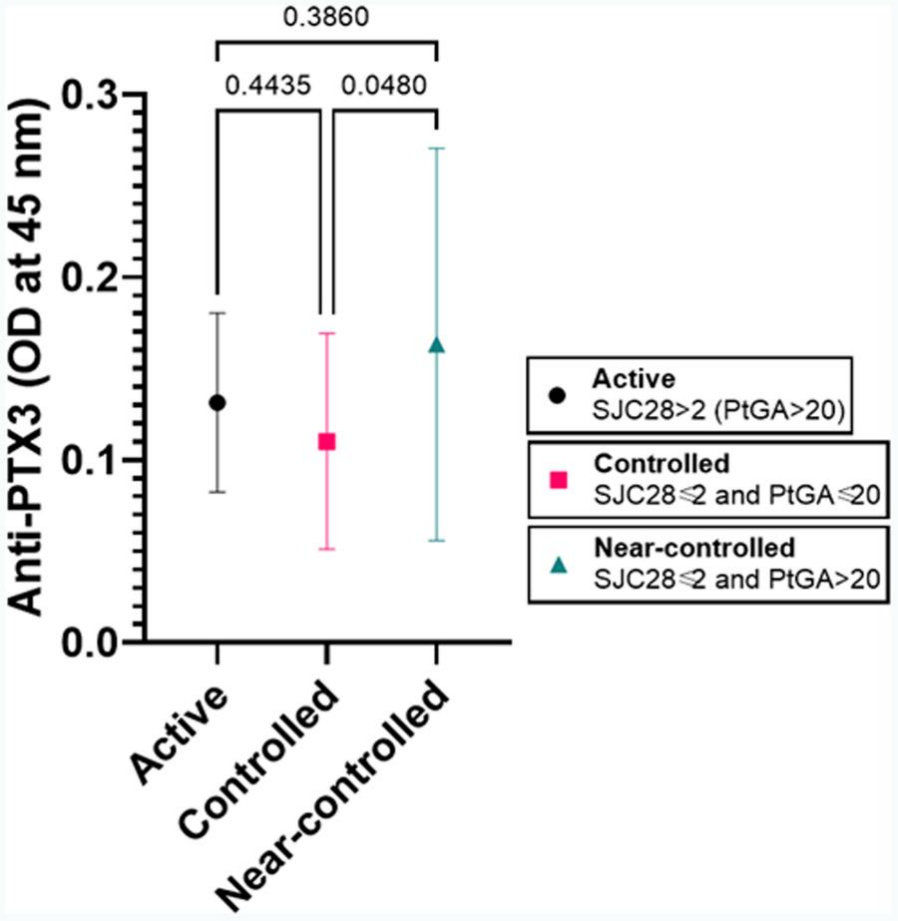
Anti-PTX3 antibodies are higher in patients with near-controlled than profoundly controlled RA, with titres comparable to patients with objectively active RA. Brown-Forsythe ANOVA *P* = 0.0187; adjusted *P* = 0.0480

At the population level, the titres of anti-PTX3 antibodies were not modified by the patient’s age or disease duration. As we expected, we found a significant correlation between anti-PTX3 titres and CDAI (*r* = 0.255, *P* = 0.022). However, it was primarily sustained by a correlation between anti-PTX3 antibodies and the CDAI subcomponent PtGA (*r* = 0.257, *P* = 0.020), while there was no significant correlation between anti-PTX3 antibodies and TJC28, SJC28, or PhGA. Moreover, anti-PTX3 antibodies were positively correlated with other PROMs, with higher titres correlated with worse scores of VAS-pain (*r* = 0.233, *P* = 0.035), PtGH (*r* = −0.235, *P* = 0.044), HAQ (*r* = 0.311, *P* = 0.015; *n* = 61), while there was an inverse relationship with FACIT-Fatigue (*r* = −0.311, *P* = 0.016; *n* = 60) (Fig. 1). Patients with low SJC28 and high PtGA had significantly higher anti-PTX3 antibodies than patients with low SJC28 and low PtGA, but not higher than patients with high SJC28 (Brown-Forsythe ANOVA *P* = 0.019; adjusted *P* = 0.048; Fig. 2). Anti-PTX3 antibodies were inversely correlated with RF (−0.232, *P* = 0.034) but not ACPA (*r* = 0.073, *P* = 0.514) titre. Anti-PTX3 antibodies did not show any significant correlation with CRP, ESR or other laboratory values. In multivariable regression analysis, anti-PTX3 antibody titres were confirmed to be significantly associated with PtGA independently of the patient’s BMI, dose of prednisone, swollen joints, and ESR (Table 2).

**Table 2.** Multivariable regression showed a significant linear association between anti-PTX3 antibodies and PtGA, independent of other clinical predictors

Predictor of PtGA	β	95% CI for β	*t*	*P*-value
Anti-PTX3 titre	0.206	0.4, 23.7	2.1	**0.042**
BMI	0.155	−0.4, 2.6	1.5	0.136
Prednisone mg daily	0.266	0.8, 5.4	2.6	**0.010**
SJC28	0.111	−6.2, 19.6	1.0	0.305
ESR	0.198	−0.5, 13.7	1.8	0.069

Values in bold are statistically significant.

### Anti-PTX3 antibodies in seropositive and seronegative RA patients

**Figure 3.**
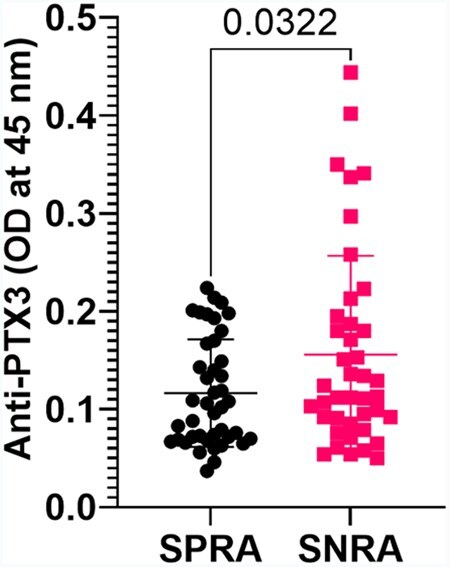
Anti-PTX3 antibodies are more prevalent in seronegative RA patients. Bullets and squares depict individual values. The mean and S.D. are represented by horizontal lines. *P*-values refer to two-tailed unpaired *t*-test with Welch correction. SNRA: seronegative rheumatoid arthritis; SPRA: seropositive rheumatoid arthritis

At group level, the mean titres of anti-PTX3 antibodies were significantly higher in seronegative compared with seropositive RA patients (*P* = 0.0322; Fig. 3), and values above the cut-off for SLE (>0.234 nm) were significantly more prevalent in seronegative (8/41) than seropositive (0/42) RA patients (19.5% vs. 0%, *P* = 0.002). However, anti-PTX3 antibodies were not associated with serostatus independently of PtGA (OR 0.57, 95% CI 0.22, 1.46; *P* = 0.242), meaning that higher PtGA in seronegative RA patients was the main reason for more prevalent anti-PTX3 antibodies in this subgroup.

### Anti-PTX3 antibodies in RA compared with FM and PsA

**Figure 4.**
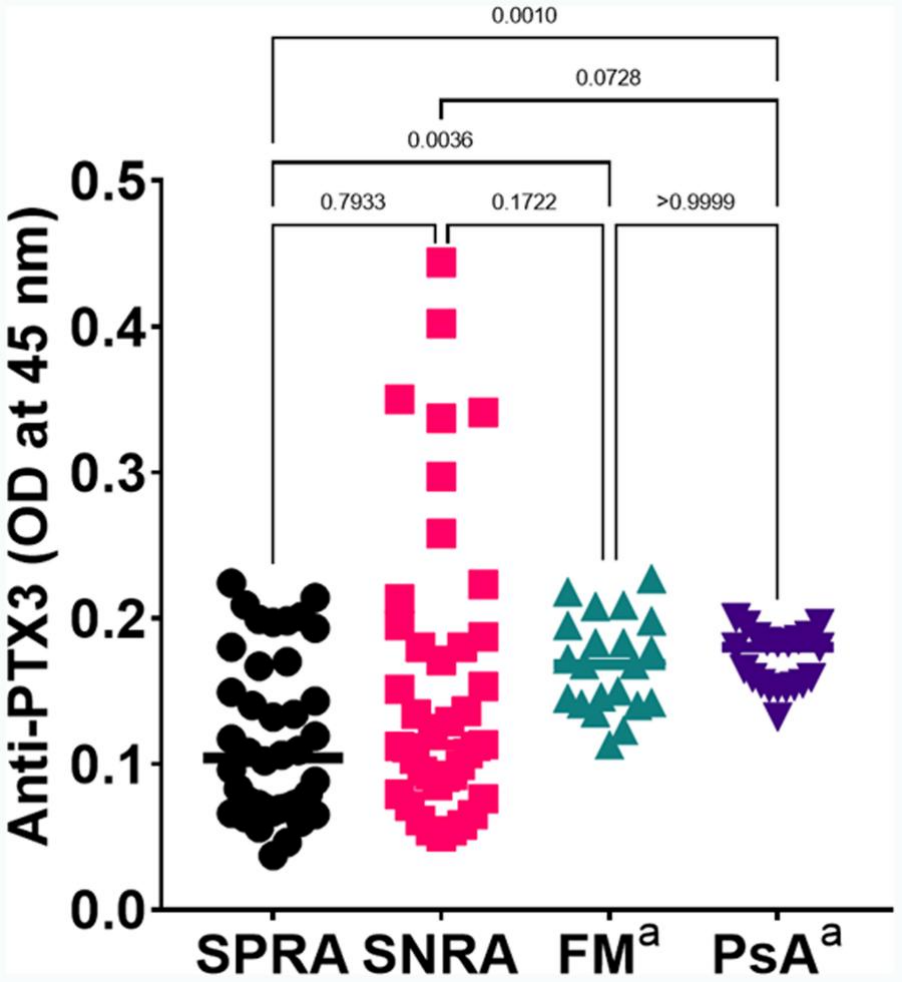
Anti-pentraxin-3 (anti-PTX3) antibody serum titres in unmatched patients with RA (SPRA, *n* = 42, SNRA, *n* = 41), FM (*n* = 21), and PsA (*n* = 21). Horizontal black lines represent the median for each group, symbols are individual data. *P*-values adjusted for multiple comparisons between groups are shown (Kruskal−Wallis test with Dunn’s multiple comparison tests, ANOVA *P* = 0.0003). ^a^Data retrieved from Bassi *et al*. [10]

We compared titres of anti-PTX3 in the present RA cohort with historical controls with FM or PsA from our previous study in 2010 (Fig. 4). Patients with seropositive RA in our study had significantly lower titres of anti-PTX3 antibodies compared with FM (0.120 ± 0.063 vs. 0.169 ± 0.032, *P* = 0.0048) and PsA (0.120 ± 0.063 vs. 0.171 ± 0.020, *P* = 0.0014) patients, while there were no significant differences between seropositive and seronegative RA, or seronegative RA and FM or PsA patients. However, we could not correct this analysis for PtGA as this data was not available for historic controls.

### Effects of DMARDs on anti-PTX3 antibodies

The mean titres of anti-PTX3 antibodies were similar in patients treated with csDMARDs or b/tsDMARDs. However, among RA patients treated with b/tsDMARDs, there was a significant effect of both treatment (*P* = 0.022) and PtGA (*P* = 0.005) on anti-PTX3 antibodies, with mean titres significantly higher (*P* = 0.025) in patients treated with TNFi (0.135, *SE* = 0.911; reference) compared with non-TNFi bDMARDs (abatacept + tocilizumab; 0.092, *SE* = 0.897) and JAKi (0.131, *SE* = 0.868) (Supplementary Fig. S1, available at *Rheumatology* online). However, when we corrected the analysis for PtGA (as an interaction term with b/tsDMARD), mean anti-PTX3 titres were significantly higher in TNFi and JAKi users, but not in users of non-TNFi bDMARDs, meaning that non-TNFi bDMARDs could effectively reduce anti-PTX3 titres. The mean titres of anti-PTX3 antibodies were similar in patients on b/tsDMARDs treated with or without MTX; likewise, anti-PTX3 titres were unaffected by the concomitant dose of MTX or oral glucocorticoids.

## Discussion

The main finding of our study was that anti-PTX3 antibodies were enriched in RA patients with high self-reported disease activity, despite evidence of clinically inactive synovitis.

Anti-PTX3 antibodies were significantly associated with pain and patient-reported outcomes of RA disease activity, despite an efficient suppression of inflammation. Indeed, extra-articular manifestations of RA, which typically develop in patients with high RF/ACPA, were also less common in anti-PTX3+ patients, and anti-PTX3 levels were inversely associated with RF titres. Overall, our findings confirm the anti-inflammatory scavenger role of anti-PTX3 antibodies. Even more interestingly, anti-PTX3 antibodies were not associated with signs of local inflammation (SJC28), yet they were significantly associated with patient’s pain, global health, and disability. One explanation is that anti-PTX3 could reduce both the systemic and local inflammatory response. Tolerance breakdown mechanisms for PTX3 might happen due to molecular mimicry or activation of a bystander clone in the context of danger and damage [18]. Since NETosis is enhanced in SLE, AAV, and RA, one hypothesis is that NETs may act as a scaffold for autoantigen presentation [19]. Our group demonstrated that anti-PTX3 antibodies reduced the burden of PTX3 deposition in the kidney and counteracted nephritogenic antibodies in lupus-prone mice [12]. It has been shown that the synovial expression of PTX3, which is the target of anti-PTX3 antibodies, is increased in RA, and it has been associated with higher disease activity and a lympho-myeloid pathotype [5]. Herein, we hypothesize that anti-PTX3 antibodies may exert anti-inflammatory effects in RA. PTX3, an essential component of innate immunity, is elevated in RA and preferentially bound to CD14+ monocytes. The complement fragment C1q is essential in promoting binding and results in enhanced release of inflammatory cytokines, including TNF-α, IL-1β, and IL-6 [7]. One possibility is that anti-PTX3 antibodies may bind C1q and prevent cytokine release, hence being associated with the beneficial anti-inflammatory effects of DMARDs. However, our study expands the comprehension of disease activity control in RA. Higher levels of anti-PTX3 antibodies could help disentangle RA disease activity assessment, which is often difficult in the setting of an unexplained discrepancy between high PtGA and low synovitis scores [20–22]. Indeed, RA patients with low SJC28 but high PtGA had significantly higher anti-PTX3 antibodies than those with both low SJC28 and low PtGA, but their levels were not dissimilar to levels found in patients with high SJC28 (all with high PtGA), revealing that such a group of patients with high levels of self-reported pain despite small evidence of inflammation represent true disease activity and not just secondary painful syndromes.

Our previous study investigated the prevalence of anti-PTX3 antibodies in a cohort of patients with various rheumatic musculoskeletal diseases, including ACPA-positive RA patients, as well as healthy and disease controls [10]. In this study, we expanded the population to include seronegative RA patients to comprehensively characterize anti-PTX3 antibodies across the RA spectrum. This design enabled us to demonstrate that mean levels of anti-PTX3 antibodies were generally lower in most RA patients compared with those with FM and PsA, which may reflect better overall disease control and quality of life in RA [23]. However, a subset of seronegative RA patients exhibited markedly elevated anti-PTX3 levels. Our findings suggest that higher anti-PTX3 antibody levels can help identify true active disease in RA patients with minimal objective signs of arthritis, particularly among seronegative RA patients. This indicates that this subset might be undertreated due to unrecognized disease activity.

Finally, the prevalence of anti-PTX3 antibodies in RA could be higher than reported in our cohort. In a larger monocentric cohort from China of untreated RA patients (*n* = 559) [1], anti-PTX3 prevalence was higher than in our sample (27% vs. 8.4%), while the proportion of anti-PTX3-positive patients was similar, independent of ACPA status (27.17% in ACPA+ vs. 25.56% in ACPA−). Also, higher values of anti-PTX3 were observed in both groups compared with our cohort. Although we found similar mean levels of anti-PTX3 antibodies in our seropositive and seronegative RA patients, the highest values were almost exclusive of seronegative RA. One reason for this difference could be the effect of treatment on the anti-PTX3 antibody titre. Li *et al.* only included treatment-naive patients or those that had been off DMARDs for at least 3 months, whereas we included RA patients on active therapy with b/tsDMARDs and/or csDMARDs. Compared with patients receiving csDMARDs only, patients on b/tsDMARDs had similar levels of anti-PTX3 antibodies. However, we found that patients treated with tocilizumab or abatacept had lower anti-PTX3 levels than those treated with TNFi or JAKi when PtGA was taken into account. This might suggest an effect of indirect B cell modulation on antibody production or the modulation of pain/PtGA seen with inhibition of IL-6 [24, 25] compared with TNFi.

### Strengths and limitations of the study

This study’s primary strength lies in its comprehensive examination of anti-PTX3 antibodies across the spectrum of RA, including both seropositive and seronegative patients. The inclusion of well-characterized historical controls with PsA and FM enhances the robustness of our comparative analyses. The use of validated and standardized in-house ELISA for antibody measurement ensures reliability and reproducibility of the results. Additionally, the detailed collection of demographic, clinical, and PROMs allows for a thorough investigation of the associations between anti-PTX3 antibodies and disease activity.

However, the study also has several limitations. The sample size is relatively small, which may affect the generalizability of the findings. The cross-sectional design precludes the ability to infer causality between anti-PTX3 antibody levels and disease activity. Furthermore, the lack of a longitudinal follow-up limits our understanding of how these antibody levels change over time with disease progression and treatment. The absence of a control group of healthy individuals in this specific study is another limitation, although previous work by our group has addressed this gap. Lastly, the reliance on serum measurements without corresponding synovial fluid or tissue analysis restricts our ability to draw definitive conclusions about the local effects of anti-PTX3 antibodies in the synovium.

## Conclusions

Our study provides novel insights into anti-PTX3 antibodies in RA. Our study reveals that elevated anti-PTX3 antibody levels are associated with residual active disease in RA patients, particularly among those who are seronegative. These findings highlight the potential of anti-PTX3 antibodies as a biomarker for detecting true disease activity, which may aid in the better management and treatment of RA, ensuring that patients with overlooked disease activity receive appropriate care.

## Supplementary Material

keae529_Supplementary_Data

## Data Availability

The data presented in this study are available upon request from the corresponding author.
