# Supplementary material for: Anti-pentraxin 3 antibodies and residual disease activity in rheumatoid arthritis
Source: Rheumatology (Oxford). 2024 Sep 28;64(4):1672–8. doi: 10.1093/rheumatology/keae529 (PMC11962886; doi:10.1093/rheumatology/keae529)
Supplement: keae529_Supplementary_Data [file keae529_supplementary_data.docx]

**Supplementary material - RHE-24-1202.R1**


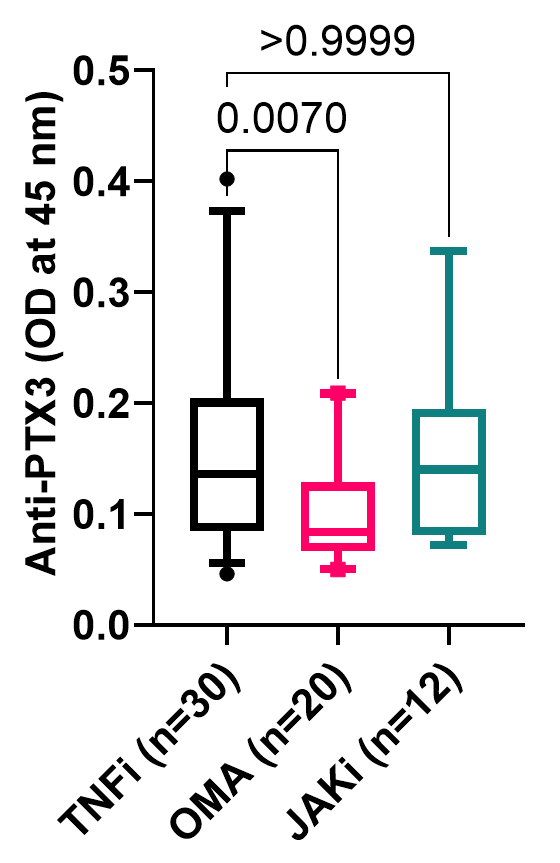


**Supplementary Figure S1. Anti-PTX3 antibody serum titres in patients with rheumatoid arthritis according to the b/tsDMARD used.** Anti-PTX3, anti-pentraxin-3 antibodies; TNFi, tumor necrosis factor inhibitor; OMA, other mechanism of actions; JAKi, janus kinase inhibitor. Box and whiskers plot with 95% confidence intervals. P-values refer to Brown-Forsythe and Welch ANOVA tests with Dunnett’s T3 multiple comparisons tests (reference category: TNFi).
